# Selection of reference genes suitable for normalization of qPCR data under abiotic stresses in bioenergy crop *Arundo donax* L.

**DOI:** 10.1038/s41598-017-11019-0

**Published:** 2017-09-06

**Authors:** Michele Poli, Silvio Salvi, Mingai Li, Claudio Varotto

**Affiliations:** 1 Department of Biodiversity and Molecular Ecology, Research and Innovation Centre, Fondazione Edmund Mach, Via E. Mach 1, 38010 S. Michele all’Adige (TN), Italy; 2 Department of Agricultural Sciences, University of Bologna, Bologna, Italy

## Abstract

Suitable reference gene selection in qRT-PCR is a key pre-requisite to produce reliable data in gene expression analyses. In this study, novel primers for six commonly used reference genes (*AC1*, *TLF*, *Act2*, *TUB α*, *EF-1α* and *GAPDH*) plus two new candidates (*pDUF221* and *RPN6*) were designed and comparatively tested for expression stability under abiotic stresses (osmotic, heavy metal and heat shock) in shoot, root and their combination of *Arundo donax* L., a raising non-food energy crop. Expression stability rankings from the most to the least stable gene in each condition and in two tissues (young shoots and roots) were generated with geNorm, NormFinder and BestKeeper programs. All programs provided similar rankings and, strikingly, in most cases identified one of the new candidates, *RPN6*, as the most suitable reference gene. This novel set of reliable references allows to choose either the best combination of reference genes across multiple stress/organ conditions or to select condition-specific genes that can improve the quality of qRT-PCR analysis. This work provides a solid basis for the functional characterization of *A. donax*, by enabling accurate quantification of the transcriptional responsiveness under a series of common stress conditions of any gene of interest in this promising biomass/bioenergy species.

## Introduction

Reverse transcription - quantitative polymerase chain reaction (RT-qPCR) is a well-established and widely used technique for gene expression analyses in many biological fields. Its precision and sensitivity allow to accurately measure the transcriptional variations of a gene among different samples, providing basic information on its function through the characterization of its tissue- or stress-specific expression pattern. Two methods for quantification are commonly used in qRT-PCR experiments: absolute and relative quantification. The absolute quantification identifies the exact copy number of transcribed RNA in a given sample. This method relies on a pre-built calibration curve that associates the known concentrations of cDNA template standards with the corresponding fluorescence data produced during RT-PCR amplification. Fitting the real-time PCR data from unknown samples to the calibration curve, thus, provides the extrapolated absolute number of copies of target gene. In addition, by normalizing the copy numbers of target gene with that of reference gene, it could also provide relative quantification comparisons between samples^1^. Relative quantification compares the gene of interest with an internal reference gene to obtain the expression variation^2^. This method requires a gene (or multiple genes) with stable expression levels as calibrator to be compared with the gene of interest in order to eliminate possible sources of errors/differences in sample preparation (e.g. RNA extraction, quantification, reverse transcription). Between these two methods, relative quantification has broader applications. It is not only used to assess differential expression of target genes in different tissues/cells, in test conditions with respect to a control condition^3^ and in allelic discrimination^4^, but it is also widely used to confirm microarray and transcriptomic data^5, 6^. Despite many advantages and applications, the most critical aspect of relative quantification is the choice of the internal control, that, ideally, should be expressed at constant level in every condition and tissue/cell type^7^. In plants, several widely used reference genes play a role in basic cellular structure and basic metabolism, like 18 S rRNA (18 S ribosomal RNA), 28 S rRNA (28 S ribosomal RNA), *ACT* (Actin), *EF-1α* (Elongation Factor-1α),*GAPDH* (glyceraldehyde-3-phosphate dehydrogenase), *TUB-α* (alpha-tubulin), *TLF* (translation factor), *RPII* (RNApolymerase II). Nevertheless, many evidences clearly indicated that their basic functions do not exclude possible transcriptional variability among conditions or growing stages, therefore, each reference gene needs to be proven adequate case by case^8^. Until few years ago only few commonly used plant reference genes were available due to limited sequence information for the majority of non-model species. With the advent of next generation sequencing technologies, however, large-scale transcriptome analysis can provide tens to hundreds of possible reference genes for any species of interest at accessible costs. RNA-Seq technology allows to simultaneously detect virtually all the expressed transcripts of a plant sample, providing at the same time also estimates of their expression levels^9^. The choice of the best reference genes for the specific set of samples, however, is not straightforward and to help in this task many algorithms have been developed. The most widely used ones are geNorm^10^, NormFinder^11^, BestKeeper^12^, RefFinder^13^ and Delta Ct^10, 14^. While most of these tools are equivalent from many points of view, not all of them take into account the same parameters of qRT-PCR. For example, RefFinder is an online tool that calculates a rank for the other four algorithms but it does not take into account the efficiency of each primer pair, assuming it *a priori* approximately 100%^15^. Similarly, delta Ct is based on pairwise comparison of raw Cq values among candidates without efficiency correction, which in case of variable amplification efficiencies could lead to biased results.

*Arundo donax* L. (commonly known as giant reed) is a fast-growing grass that belongs to the Poaceae family. Its high biomass production (up to 40 tons/ha) and low input requirements, make it one of the best options as non-food energy crop, especially in the Mediterranean area where it is naturalized since thousands of years^16^. Several studies characterized the major agronomic features of this crop, focusing mainly on the sustainability of its cultivation in different conditions or environments^17–20^, especially in marginal lands^21^. *A. donax* was also shown to potentially be an important species in remediation of soil contaminated by heavy metals^22^. With the recent development of transcriptomic resources for this non-model species^23–25^ a large number of gene expression data is now available for further functional characterization. To date, however, no validated set of reference genes specific for expression studies in *A. donax* under stressed conditions is available.

To fill this important gap in the functional genomics toolbox of *A. donax*, in this study eight candidate reference genes with diverse functional categories were selected to assess their expression stabilities in two different organs (shoots and roots) of young seedlings subjected to three different stress treatments: osmotic stress (15% PEG 6000), heavy metal (500 µM CdSO_4_) and heat shock (42 °C). Among these candidates, six have been previously used as reference genes in RT-qPCR analyses while the other two were newly selected from transcriptome analyses of giant reed and sorghum, respectively. Finally, to validate the reliability of candidate reference genes, we evaluated their performance under osmotic, heat and heavy metal stress. For the first two stresses we used *A. donax DREB2A* (Dehydration-Responsive Element Binding Protein 2 A)^26^, a well-known drought- and heat-responsive gene. In addition, we also confirmed with the two most stable and the two least stable among our new reference genes the expression pattern of *IspS* (isoprene synthase), a hemiterpene synthase which is transcriptionally regulated in response to stress induced by the heavy metal cadmium^27^.

## Results

### Gene selection, amplification specificity and efficiency

In this study, we selected eight candidate reference genes from different sources. Four of them, namely *TLF* (*translation factor*), *Act2* (*actin2*), *Tub α* (*alpha tubulin*) and *EF-1 α* (*elongation factor 1-alpha*), have been already used in qRT-PCR experiments in foxtail millet^28^, the species with a sequenced genome which is most closely related to *A. donax* ^23^. For all these genes, the *A. donax* homologs which were used for primer design shared a nucleotide similarity greater than 91% to foxtail millet references. Published primers for foxtail millet were slightly adapted to giant reed with exception of TLF for which completely new primers were designed (Table 1, Supplementary Table S1). By mining the sorghum transcriptome^29^, we extracted two more candidates: the commonly used *GAPDH* (*glyceraldehyde-3-phosphate dehydrogenase 2*) and *RPN6* (*26 S proteasome non-ATPase regulatory subunit 11*). *A. donax* homologs share respectively 91% and 92% of sequence similarity to their closest sorghum homologs (Supplementary Table S1). The other candidate gene homologous to an *Actin* gene (*AC1*) in sorghum (having 89% similarity at nucleotide level) used in the previous study^24^ was included in this analysis. Finally, one new gene *pDUF221* has also been considered as a candidate based on transcriptomic data analysis in *A. donax* ^24^. To determine the amplification specificity of each primer pair prior to intensive qPCR analysis, electrophoretic analysis on agarose gel and melting curve assessment were carried out for each amplification; a single amplification band with expected size and length for each primer pair was observed on 2% agarose gels, and a single peak was detected through melting curve analysis for all amplifications and no signal for non-template control (Supplementary Figure S1). Moreover, for all primer pairs, amplification efficiencies were calculated based on standard curve assay generated from amplifications with a series of cDNA dilutions and resulted to be between 92.85% and 104.03% with correlation coefficients (R^2^) ranging from 0.987 to 0.998 (Table 1, Supplementary Figure S2).

**Table 1:** Summary of the eight candidate reference genes tested and two *A. donax* target genes used for their validation. Sequence and amplicon characteristics are provided for each primer pair. Underlined primers/bases are specifically designed for *A. donax*. E (%) is primer efficiency calculated with standard curves and formula E = 10 ^(−1/slope)^*100; R^2^ is the correlation coefficient; Tm is the melting curve temperature. Asterisc (*) indicates the target genes used for reference gene validation.

**Gene symbol**	**Gene Name**	**Accession No**.	**Primer Pair (5′-3′)**	**Product size (bp)**	**E (%)**	**R** ^**2**^	**Tm (°C)**	**Ref**
AC1	Actin	Unigene036290	F: TCTTGGCTTGCATTCTTGGG	93	100,72	0,998	81,5	24
			R: TGGATTGCGAAGGCTGAGTAC					
Act2	Actin 2	Unigene057037	F: CGCATACGTGGCACTTGACT	126	92,85	0,987	83,5	28
			R: GGGCATCTGAACCTCTCTGC					
EF-1α	Elongation factor 1-alpha	Unigene076509	F: TGACTGTGCTGTGCTCATCA	133	97,13	0,996	83	^28^
			R: GTTGCAGCAGCAGATCATCT					
GAPDH	Glyceraldehyde 3-phosphate dehydrogenase	Unigene069707	F: TGACAAGGAGAAGGCTGCTG	167	103.83	0.997	82.5	^29^
			R: GAGCAAGGCAGTTTGTGGTG					
pDUF221	Probable membrane protein DUF221-related Calcium-dependent channel	Unigene070087	F: GACAAAGGAGTCAGCCGTCA	91	99.94	0.998	81	^24^
			R: AACGTGCTTCGGACTTGGAT					
RPN6	26 S proteasome non-ATPase regulatory subunit 11	Unigene067565	F: CACACGACTAGCAGCTTTCAAG	78	104.03	0.993	80	^29^
			R: TTCAAACGTCGGGAAGGTTG					
TLF	Translation Factor	Unigene076539	F: GACTTCATGGGTGGTGCTGA	110	100.32	0.998	80	^28^
			R: TGTTTGTTGGGGGACTTGCT					
TUB α	Tubulin alpha	Unigene068813	F: TACCAGCCACCCTCAGTTGT	121	96.24	0.996	85	^28^
			R: AGTCGAACTTGTGGTCAATGC					
DREB2A*	Dehydration-Responsive Element Binding Protein 2	Unigene057213	F: TCCAGCAGGTAGATCATCTCC	98	98.59	0.999	78	^24^
			R: AGCAGGTTCGGTAATAGGCA					
IspS*	Isoprene Synthase	KX906604	F: GAGGTTCCGTTGCATTTGAGR: CAAGAGCAACATCTGTCCAC	184	99.31	0.987	80.5	^27^

### Gene expression profile of candidate reference genes

**Figure 1:**
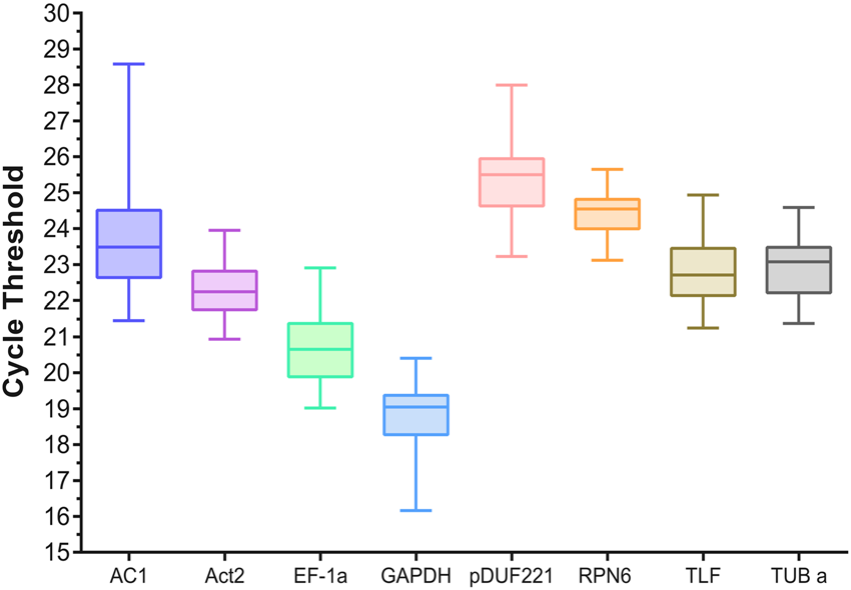
Expression level variability of each candidate reference gene. Boxes extend from the 25^th^ to 75^th^ percentiles, whiskers represent the maximum and minimum values, the line across the box represents the median value of the Cq values for each gene.

To assess the transcriptional variation and stability of each candidate reference gene, cycle threshold (Cq) value was obtained using qPCR to estimate the expression level of each gene among all the samples (two *A. donax* tissues for three stress conditions, each with five time points plus one pre-stress control) (Supplementary Table S2). The highest Cq value was detected for *AC1* (lowest expression: 28.58 cycles), while the lowest Cq value was measured for *GAPDH* (highest expression: 16.16 cycles). Mean expression values per gene varied from 25.28 of *pDUF221* to 18.84 of *GAPDH*. To provide a more informative stability index, we calculated also the difference between 75^th^ and 25^th^ percentile (ΔP), which was inversely proportional to the spread of the data^30^. Based on this criterion, *RPN6* (ΔP = 0.82) was the most stable gene, followed by *Act2* (1.07), *GAPDH* (1.01), *TUB α* (1.27), *pDUF221* (1.31), *TLF* (1.32), *EF-1α* (1.48) and *AC1* (P = 1.87) (boxes in Fig. 1). This stability ranking among genes is also confirmed by the coefficient of variation (CV) that ranged from 2.5% of *RPN6* to 6.24% of *AC1* (Supplementary Table S3). Comparison of the expression profile of each gene from 0 (control) to 24 h after stress application (Supplementary Figure S3) showed that *GAPDH* was always the most highly expressed gene in every condition/tissue. The lowest expression levels were mainly associated to *pDUF221*, with the exception of heat shock stress. Only for this stress, in shoot *RPN6* had higher Cq at 3 h and 6 h and, in root, *RPN6* and *AC1* had higher Cq values for the whole time course (Supplementary Figure S3).

### Overall expression stability analyses of candidate reference genes under stress treatments

All treatments, divided according to tissue, were included in an overall analysis to understand which candidate gene is the most stable across samples. For shoot tissue, the results were heterogeneous, with a prevalence of *EF-1α* and *RPN6* in the top three genes for all the algorithms (Table 2). In this case, geNorm indicated *TLF* and *TUB α* as the most stable genes, while they were ranked as sixth and fourth by NormFinder and as last and fifth by BestKeeper. The least stable genes were instead consistently *AC1* and *pDUF221* with the only exception of BestKeeper that identified, as mentioned above, *TLF* as the worst ranking gene. Also, in root *EF-1α* and *RPN6* genes were always in the top three positions (with the exception of *Act2* ranked first by BestKeeper), while *pDUF221* and *AC1* were relegated strictly to the last two ranks by all algorithms. Taking into account both shoot and root organs, *RPN6* appeared as the candidate gene with the best scores in the three algorithms followed by *EF-1α* (first, second and sixth) (Supplementary Table S4). Overall intergroup and intragroup analyses were also carried out for all samples with NormFinder. This analysis graphically showed the variation of each gene with respect to four subgroups: control, osmotic, heavy metal and heat shock stresses (Supplementary Figure S4). Analysis of the best combination of two genes was consistent with single stability value in root, where *EF-1α* + *RPN6* (combination value of 0.105) was the best pair of genes. More surprisingly, in shoot the best pair of genes suggested were *RPN6* (second, S = 0.188) and *TLF* (sixth, S = 0.320) with a combination value of 0.091. It should be noticed that also geNorm algorithm included *TLF* in the best pair of primers together with *TUB α*. Considering together shoots and roots, NormFinder suggested the combination between *RPN6* and *GADPH* with a value of 0.115 (the latter classified at the sixth position as a single gene) (Supplementary Figure S4C). These differences with respect to the results of the gene by gene analyses were due to the fact that NormFinder tended to enhance intergroup stability by balancing over/under expression of the two genes to be as close as possible to zero in each subgroup.

**Table 2:** Stability ranking of each candidate reference gene using different algorithms in shoot and root. Column “value” corresponds to M in geNorm, S in NormFinder and CV ± SD in BestKeeper.

**Rank**	**Shoot**						**Root**					
	**GeNorm**		**NormFinder**		**BestKeeper**		**GeNorm**		**NormFinder**		**BestKeeper**	
	**Gene**	**Value**	**Gene**	**Value**	**Gene**	**Value**	**Gene**	**Value**	**Gene**	**Value**	**Gene**	**Value**
**1**	TLF TUB α	0.421	EF-1α	0.132	RPN6	1,00 ± 0,25	EF-1α RPN6	0.353	RPN6	0.111	Act2	2,05 ± 0,45
**2**	EF-1α	0.480	RPN6	0.188	GAPDH	2,00 ± 0,39	TLF	0.409	EF-1α	0.129	EF-1α	2,40 ± 0,48
**3**	RPN6	0.559	TUB α	0.232	EF-1α	2,15 ± 0,46	TUB α	0.477	TLF	0.234	RPN6	2,02 ± 0,49
**4**	GAPDH	0.626	Act2	0.234	Act2	2,70 ± 0,61	GAPDH	0.571	TUB α	0.270	TLF	2,65 ± 0,60
**5**	Act2	0.655	GAPDH	0.245	TUB α	2,69 ± 0,62	Act2	0.636	Act2	0.306	GAPDH	3,44 ± 0,63
**6**	AC1	0.700	TLF	0.320	pDUF221	2,69 ± 0,69	pDUF221	0.840	GAPDH	0.421	TUB α	2,76 ± 0,64
**7**	pDUF221	0.792	AC1	0.470	AC1	3,08 ± 0,72	AC1	1.012	pDUF221	0.783	pDUF221	3,13 ± 0,78
**8**			pDUF221	0.568	TLF	3,32 ± 0,77			AC1	0.819	AC1	5,82 ± 1,41

### Expression stability analyses of candidate reference genes under water/osmotic stress treatment

Water stress was induced by adding 15% of PEG 6000 to the hydroponic solution which decreased the osmotic pressure of the media, consequently inducing osmotic and water limitation stress in the plants^31^ without toxic effects in the short term^32^. Reference gene analyses under this simulated drought condition in *A. donax* shoots showed a high and shared rank for *RPN6* (first in NormFinder and BestKeeper, second in geNorm) followed by *EF-1α* gene (first in geNorm, fourth in NormFinder and third in BestKeeper) (Table 3). In roots, *RPN6* was confirmed as the most stable gene followed by *GAPDH* that had the best value in geNorm (shared with *RPN6*) and BestKeeper. Using both organ’s samples, *RPN6* and *GADPH* were the best two genes (Supplementary Table S4), resulting in the top three ranks provided by all programs. The lowest stability values resulted from the *AC1* and *pDUF221* genes with all algorithms and tissues with exception of BestKeeper that ranked *pDUF221* at the fourth position in root and *TLF* and *EF-1α* as last in the shoot + root analysis.

**Table 3:** Stability ranking of each candidate reference gene using different algorithms under osmotic stress in shoot and root. Column “value” corresponds to M in geNorm, S in NormFinder and CV ± SD in BestKeeper.

**Rank**	**Shoot**						**Root**					
	**GeNorm**		**NormFinder**		**BestKeeper**		**GeNorm**		**NormFinder**		**BestKeeper**	
	**Gene**	**Value**	**Gene**	**Value**	**Gene**	**Value**	**Gene**	**Value**	**Gene**	**Value**	**Gene**	**Value**
**1**	TLFEF-1α	0.293	RPN6	0.119	RPN6	1,16 ± 0,29	RPN6GAPDH	0.266	RPN6	0.072	GAPDH	1,73 ± 0,32
**2**	RPN6	0.345	TUB α	0.141	GAPDH	1,51 ± 0,30	EF-1α	0.308	EF-1α	0.113	RPN6	1,37 ± 0,33
**3**	TUB α	0.377	TLF	0.208	EF-1α	1,87 ± 0,40	TLF	0.392	GAPDH	0.203	EF-1α	2,22 ± 0,44
**4**	GAPDH	0.436	EF-1α	0.310	TUB α	1,73 ± 0,41	Act2	0.457	TLF	0.257	pDUF221	1,87 ± 0,47
**5**	Act2	0.485	GAPDH	0.359	TLF	1,75 ± 0,42	TUB α	0.504	Act2	0.261	Act2	2,53 ± 0,56
**6**	AC1	0.527	Act2	0.365	Act2	2,02 ± 0,47	pDUF221	0.605	TUB α	0.264	TLF	2,58 ± 0,57
**7**	pDUF221	0.656	AC1	0.603	AC1	2,50 ± 0,59	AC1	0.705	AC1	0.665	TUB α	3,45 ± 0,78
**8**			pDUF221	0.726	pDUF221	2,61 ± 0,68			pDUF221	0.676	AC1	4,75 ± 1,09

### Expression stability analyses of candidate reference genes under heavy metal stress treatment

*A. donax* is considered a suitable plant for phytoremediation of contaminated soil. Among the heavy metals that can affect soil quality and reduce plant productivity, cadmium (Cd) is one of the most toxic heavy metals^33^. *A. donax* seems able, however, to cope with it without physiological adaptation, which is an important feature for phytoremediation^22, 34^. When subjected to cadmium toxicity stress, reference gene performances were more heterogeneous respect to osmotic stress: in shoots, *RPN6* and *TUB α* ranked in the top three for all the programs, but in roots *TLF* was among the best three followed by *EF-1α* (first, second and fourth) (Table 4). On the other hand, the least stable gene in shoot was *pDUF221*, in root was *AC1* and in the full set of heavy metal stressed samples were *GADPH* and *AC1* (Table 4). Noteworthy, BestKeeper indicated as most stable candidate *pDUF221*, which was instead ranked fourth by geNorm and last by NormFinder. Interestingly, analysis of shoot and root together put *pDUF221* always in the top three positions, indicating that this gene is the most suitable across different organs for heavy metal stress treatments, but less stable if organs are taken separately (Supplementary Table 4). Other general candidate genes to be used in studies encompassing both root and shoot could alternatively be the common *Act2* (ranked fourth, second and first) and *TLF* (ranked third, first and third).

**Table 4:** Stability ranking of each candidate reference gene using different algorithms under heavy metal treatment in shoot and root. Column “value” corresponds to M in geNorm, S in NormFinder and CV ± SD in BestKeeper.

**Rank**	**Shoot**						**Root**					
	**GeNorm**		**NormFinder**		**BestKeeper**		**GeNorm**		**NormFinder**		**BestKeeper**	
	**Gene**	**Value**	**Gene**	**Value**	**Gene**	**Value**	**Gene**	**Value**	**Gene**	**Value**	**Gene**	**Value**
**1**	RPN6 GAPDH	0.303	TUB α	0.150	RPN6	1,18 ± 0,29	EF-1α RPN6	0.331	TLF	0.117	pDUF221	0,90 ± 0,23
**2**	TUB α	0.320	RPN6	0.260	GAPDH	2,12 ± 0,41	TLF	0.360	EF-1α	0.139	TLF	1,48 ± 0,33
**3**	AC1	0.358	AC1	0.311	TUB α	2,26 ± 0,51	Act2	0.388	Act2	0.160	RPN6	1,51 ± 0,36
**4**	Act2	0.392	TLF	0.324	pDUF221	2,10 ± 0,55	pDUF221	0.448	RPN6	0.162	EF-1α	2,01 ± 0,40
**5**	EF-1α	0.450	Act2	0.348	Act2	2,49 ± 0,55	TUB α	0.492	GAPDH	0.272	Act2	1,93 ± 0,43
**6**	TLF	0.489	EF-1α	0.391	EF-1α	2,59 ± 0,55	AC1	0.559	TUB α	0.273	GAPDH	2,76 ± 0,49
**7**	pDUF221	0.518	GAPDH	0.449	AC1	2,60 ± 0,58	GAPDH	0.627	AC1	0.483	TUB α	2,74 ± 0,62
**8**			pDUF221	0.737	TLF	3,02 ± 0,68			pDUF221	0.789	AC1	3,35 ± 0,78

### Expression stability analyses of candidate reference genes under heat shock stress treatment

Heat shock is an important factor that affects plant physiology and growth^35^. The capability of *A. donax* to survive in warm environments is an interesting trait of this plant^16^, which can be relevant to forecast its productivity as heat spells become more frequent. Gene stability in heat condition (42 °C) identified the *RPN6* gene as the most stable reference gene in shoot (third, first and first for geNorm, NormFinder and BestKeeper, respectively), root (first, first and second) and also considering both organs together (always first) (Table 5). Other suitable reference genes for this stress condition were *EF-1α* (first, second, second) and *TUB α* (second, third, fourth) in shoot, *EF-1α* (first, second, first) and *Act2* (third, third and fourth) in root and *TLF* (first, second, fourth) in overall heat shock (Supplementary Table S4). Again, the genes with overall lower ranking across algorithms were *pDUF221* and *AC1* in all the conditions.

**Table 5:** Stability ranking of each candidate reference gene using different algorithms under heat shock stress treatment in shoot and root. Column “value” corresponds to M in geNorm, S in NormFinder and CV ± SD in BestKeeper.

**Rank**	**Shoot**						**Root**					
	**GeNorm**		**NormFinder**		**BestKeeper**		**GeNorm**		**NormFinder**		**BestKeeper**	
	**Gene**	**Value**	**Gene**	**Value**	**Gene**	**Value**	**Gene**	**Value**	**Gene**	**Value**	**Gene**	**Value**
**1**	TLF EF-1α	0.315	RPN6	0.133	RPN6	0,95 ± 0,24	EF-1α RPN6	0.360	RPN6	0.118	EF-1α	1,92 ± 0,39
**2**	TUB α	0.367	EF-1α	0.190	EF-1α	1,72 ± 0,37	TLF	0.411	EF-1α	0.131	RPN6	1,88 ± 0,46
**3**	RPN6	0.398	TUB α	0.282	GAPDH	2,18 ± 0,42	Act2	0.488	Act2	0.219	GAPDH	2,54 ± 0,48
**4**	GAPDH	0.544	TLF	0.312	TUB α	2,11 ± 0,48	GAPDH	0.542	TLF	0.239	Act2	2,28 ± 0,50
**5**	Act2	0.599	GAPDH	0.439	Act2	2,17 ± 0,48	TUB α	0.598	TUB α	0.292	TLF	2,48 ± 0,57
**6**	AC1	0.662	Act2	0.445	AC1	2,15 ± 0,51	pDUF221	0.807	GAPDH	0.352	pDUF221	2,69 ± 0,65
**7**	pDUF221	0.745	AC1	0.484	TLF	2,42 ± 0,56	AC1	1.019	AC1	0.849	TUB α	3,23 ± 0,76
**8**			pDUF221	0.496	pDUF221	3,56 ± 0,90			pDUF221	0.851	AC1	5,51 ± 1,41

### Best reference gene number identification

**Figure 2:**
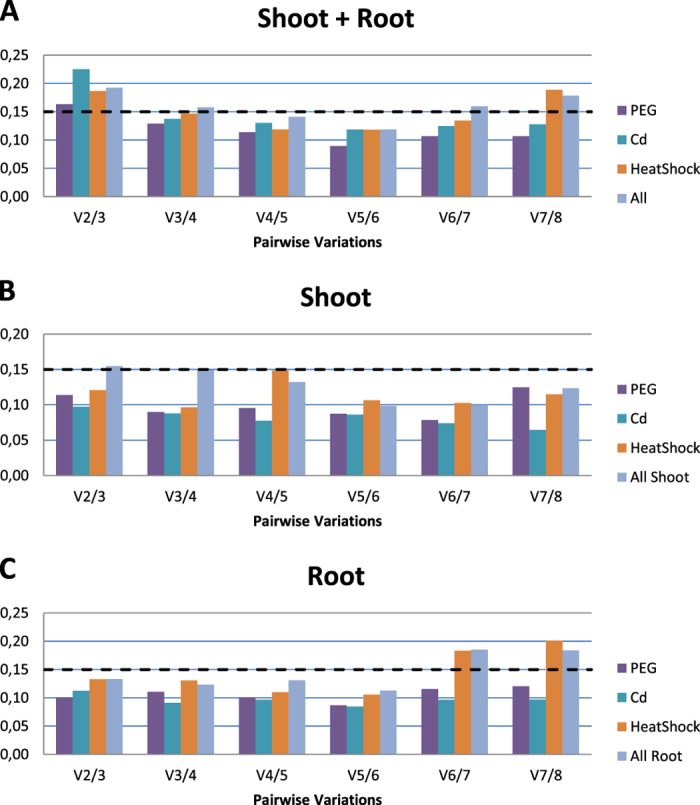
Determination of best reference gene number calculated by geNorm pairwise variation (Vn/Vn + 1) under independent stress treatment and their combination in both shoot and root together (**A**), shoot (**B**), and root (**C**).

geNorm Excel add-in is a useful tool for calculation of the best number of genes that should be used together in a relative qRT-PCR experiment. Therefore, it has been used in this study to predict the optimal number of reference genes to be used in each stress experiment (osmotic, heavy metal and heat shock). As expected, the number of references depended on the experimental settings: considering shoot and root together geNorm indicated three genes as the most suitable for single stresses and four genes considering all stresses (Fig. 2A). If root and shoot were considered separately, all the values for single stress dropped below the suggested threshold of 0.15 (Fig. 2B,C). With one exception in shoot, by grouping the three stresses, the value was just above the threshold limit when two candidate genes were used while it laid on the threshold with three.

### Validation of reference genes by expression pattern analyses of target genes under stress treatments

**Figure 3:**
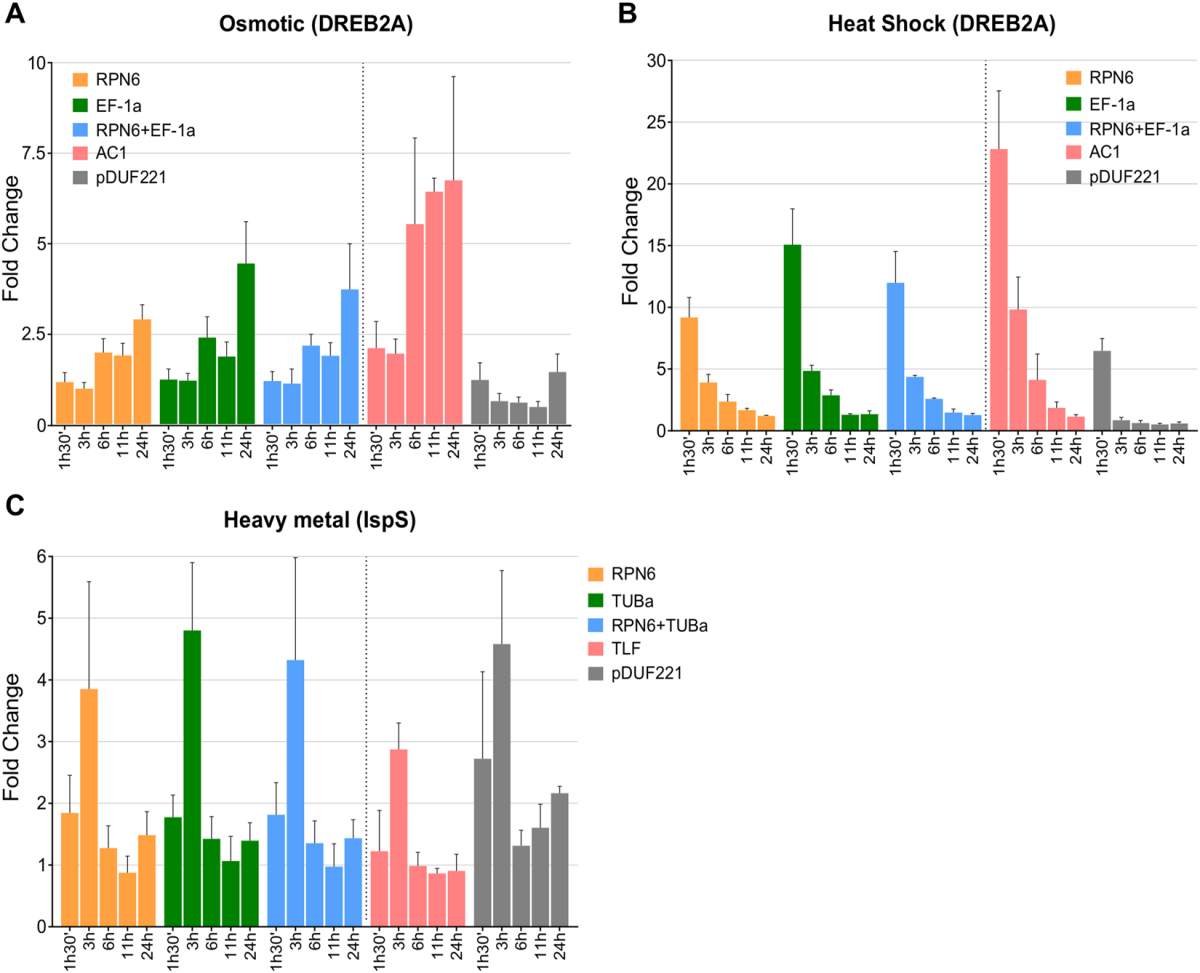
Relative expression of target genes in shoot under different durations of stress treatments using different reference genes for normalization. Relative expression of *DREB2A* under osmotic stress (**A**) and heat shock treatment (**B**), relative expression of *IspS* under heavy metal treatment (**C**). The two most stable genes, their combination (left of the vertical dotted line) and the least stable genes (right of the vertical dotted line) are used for normalization under each stress condition.

To demonstrate the reliability of the newly analysed reference genes in *A. donax* for stress treatments, *DREB2A* (*Dehydration-Responsive Element Binding Protein 2 A*) which is one of the key genes triggering the response to both drought and heat shock^26, 36, 37^ and isoprene synthase gene (*IspS*), which is differentially expressed in heavy metal (Cd) stress treatment in *Arundo donax* ^27^, were chosen to validate these reference genes for qPCR internal normalization. We compared the expression profiles of *DREB2A* in shoot under osmotic stress and heat shock treatments using the two most stable genes (*RPN6* and *EF-1 α*), their combination (*RPN6* + *EF-1 α*) and the least stable genes (*AC1* and *pDUF221*) as reference genes based on stability ranking (Tables 3 and 5, respectively). Fold change of *DREB2A* was calculated with the comparative Ct method^3^. The expression pattern was consistent with a two-fold increase of *DREB2A* expression at 6 h and 11 h and about four-fold increase at 24 h normalized with *RPN6*, *EF-1α* and their combination (Fig. 3A). On the other hand, the pattern obtained using *AC1* as reference displayed an upwards-shifted trend, with a twofold increase since the beginning and a six-fold increase from 6 h on. When *pDUF221* was used as a reference gene, the expression pattern decreased at 3 h, 6 h, and 11 h to finally grow again at 24 h. Under heat shock treatment, the expression levels of *DREB2A* normalized with *RPN6, EF-1α* and their combination were extremely consistent at all time points: after a 12-fold increase at 1 h30′, *DREB2A* expression gradually decreased to four-fold at 3 h and two-fold at 6 h. By contrast, *DREB2A* expression levels were found to be two-fold higher at 1 h30′, 3 h and 6 h using *AC1* as reference gene and at least two-fold lower at all time points using *pDUF221* compared to when using *RPN6*, *EF-1α* and their combination (Fig. 3B). Furthermore, the expression levels of *IspS* under cadmium treatment were highly consistent at all time points with a 1.8-fold increase at 1 h30′ and a four-fold increase at 3 h using *RPN6*, *TUB α* and their combination as reference genes, respectively. Meanwhile, *IspS* expression levels were shown to decrease by 30% at all time points when normalized with *TLF* compared with *RPN6*, *TUB α* and their combination. When the expression levels of *IspS* were normalization with *pDUF221*, we observed a dramatic increase. A 2.8-fold increased was observed at 1 h30′, followed by a continuous increase from 6 h till 24 h, instead of the decreased expression at 11 h and the further increase at 24 h observed when normalizing with *RPN6*, *TUB α* and their combination (Fig. 3C).

## Discussion

Real Time PCR is a powerful technique for gene expression profiling and functional characterization of genes. Its accuracy, however, is critically dependent from the choice of reference genes whose expression is strictly proportional to the total mRNA amount in the samples to be quantified^38^. Therefore, ideal reference genes should have the same expression levels (measured in terms of Cq value) among different conditions, tissues, developmental stages and crop varieties^7^. In reality, however, gene stability depends greatly on the plant-environment system so that different stress conditions, organs or cultivars can bring undesirable variations in expression of reference genes selected without specific validation, possibly leading also to erroneous quantification^8^. For this reason, a full set of possible reference genes should be developed and systematically tested for each species whenever the experimental conditions change. In this study, eight candidate reference genes were selected for *A. donax* L., an emerging non-food energy crop^39^: *AC1*, *pDUF221*, *TLF*, *Act2*, *TUB α*, *EF-1α*, *RPN6* and *GAPDH*. The aim of this study was not only to evaluate the overall performance of these genes as references for qRT- PCR data analysis, but also to provide a detailed indication on which gene is more suitable for specific organ/stress combinations. Identifying stable reference genes is not a trivial task, as it encompasses the choice of the candidates based on literature searches, the identification of the homologs from a non-model species, the design of the primers and their thorough verification in the species of interest, in this case *A. donax*. Any of these steps can fail, resulting in a considerable waste of time, effort and resources. As long as the conditions used in future studies will be the same as those used here, therefore, the relevance of our results is that they provide for *A. donax* reliable references without the need for time-consuming testing/optimiziation. Given its biological features and economic relevance^24, 25, 40^, the three kinds of abiotic stress applied in this study (osmotic, heavy metal and heat shock) are among the most interesting ones to characterize the functional bases of *A. donax* tolerance to adverse environmental conditions. Three algorithms were applied to the Cq values to measure the stability of the candidate genes: geNorm, NormFinder and BestKeeper. Our results show overall congruence on the stability ranking of the reference genes generated from different algorithms, which indicates the good performance and reliability of the methods. We also divided the analysis into stress and organ subsets to identify for each subset of samples the best performing reference genes. In general, gene *RPN6* was always ranked among the top three genes in both organs under osmotic stress, heat shock and overall analysis, which makes it a highly suitable reference (Table 2). Nevertheless, *RPN6* showed under heavy metal stress, in root and with the complete dataset slightly higher variability, especially when the NormFinder and BestKeeper algorithms were used. On the other hand, geNorm classified *RPN6* as the best reference gene also in root, making the choice difficult in this context. These results are very interesting, giving the fact that until now, this gene was considered as possible reference gene in quantitative RT-PCR only in Arabidopsis^41–43^ but, to our knowledge, never in monocot species. Its putative orthologue in Arabidopsis (*AT1G29150*) encodes a protein constituting a lid subunit of the 26 S proteasome, which is involved in the ubiquitin-proteasome system (UPS) for degradation of misfolded proteins and for stress response^44^. Other subunits of this large protease complex have been previously suggested as potential source of new and more stable reference genes in Arabidopsis^45^. Worth of notice, in Arabidopsis *RPN6* is classified as a cadmium responsive gene^46^, which could explain the variation we observed in response to this stress. We notice, however, that *RPN6* still remained more stable than some other commonly used reference gene such as *EF-1α* or *AC1* (Table 4, Supplementary Table S4), indicating a pretty limited transcriptional resposiveness of *RPN6* to cadmium in *A. donax* as compared to *A. thaliana*. This fact is possibly due to the high resistance of *A. donax* to cadmium treatment as demonstrated by Papazoglou and colleagues^34^. A useful reference gene across different stresses can be the commonly used *EF-1α*, which ranked always in the top three positions. From our analysis, this gene often scored better when the organs were considered separately, especially in osmotic and heat shock stresses (Tables 3 and 5), suggesting moderate tissue-specific variability. The *EF-1α* gene from *Setaria italica* (L.) P. Beauvois (foxtail millet) was suggested as the best internal control gene under drought and salt condition^28^. Here, we confirm the good stability of gene *EF-1α* also in *A. donax*, a close relative of *S. italica*, but mainly when used in single tissues. Considering both tissues together, the *Act2* gene performs better in each stress condition, especially under heavy metal treatment (Supplementary Table S4). *GAPDH* is, instead, suitable in osmotic stress, where it resulted the best among commonly used reference genes (Table 3). This result is consistent with previous studies showing that *GAPDH* is one of the best references in plants under drought and salt stress, but not in other conditions^47–49^. Finally, we suggest to avoid using two of the candidate genes tested due to their instability: p*DUF221* and *AC1*. In the case of *pDUF221* this result is likely due to fact that the transcriptomics analysis used for its selection encompassed a single time point only from osmotic stress, while here we tested a 24 h time series and multiple stresses. As for the number of reference genes to be used, geNorm analyses showed some interesting trends based on the different dataset employed. In general, the higher the number of organs/conditions, the higher tended to be the number of genes required for a reliable quantification. In particular, the full dataset (all the treatments + both organs) dropped below the suggested 0.15 threshold only with four reference genes, while in roots it estimated that only two genes were needed (Fig. 2A,C). This is significant because it implies a differential regulation between organs that becomes difficult to quantify in case of studies aimed at the comparison of target genes of aerial and underground tissues. Another interesting point is the marginal decrease of the pairwise variation when considering the combination of all stresses in shoot with two or three reference genes (Fig. 2B). Given the relatively high cost/benefit ratio in the use of an additional reference gene, and considering that 0.15 is an arbitrary threshold^10^, we suggest that use of two internal controls can be a good compromise for both root and shoot when interested in the organ-specific comparison of target expression levels under osmotic, heavy metal and heat stress in *A. donax*.

*DREB2A* is a well-known transcription factor, which is associated with drought, salt and heat responses. The *DREB2A* protein interacts with a *cis*-acting dehydration-responsive element and activates a downstream cascade of drought and heat-responsive genes, thus providing a better tolerance for plants to these stresses^26, 36, 37^. The consistent expression pattern obtained for *DREB2A* under osmotic and heat shock treatment normalized with the two best reference genes and highly variable pattern with the two worst ones further confirmed the reliability of the ranking of these reference genes. For the cadmium stress we used the *IspS* gene, which we recently characterized in *A. donax* and found to be transcriptionally upregulated by cadmium treatment^27^. The results of the validation indicate that, in the case of heavy metal, the performance of the reference genes for normalization between the best and least stable genes does not differ as much as in the cases of those for PEG and heat shock treatments. Given the relatively low cadmium responsiveness of *IspS*, however, it is possible that the differences in normalization among reference genes may result bigger for genes with stronger upregulation in response to cadmium.

In summary, this study provides a wide view of the reference genes that can be used or should be avoided in *A. donax* under specific abiotic stresses and in specific organs, making an important step forward towards the reliable and accurate gene expression quantification in this species. In addition, this study emphasizes further that normalization with reference genes rigorously validated before use for any new experimental design is essential. Moreover, thanks to analysis of related species transcriptomes, a new stable gene (*RPN6*) has been successfully used for relative quantification, showing that a deeper comparative analysis of plant transcriptomes can unveil additional candidates for a more precise and reliable qRT-PCR analysis. We expect that *RPN6* could find application in additional monocotyledonous species.

## Methods

### Plant materials and stress treatments

Cohorts of *A. donax* cuttings (Sesto Fiorentino, Florence, Italy 43°49′01.8′N 11°11′57.0′E) were used in this study. The plant growing condition and procedure for stress treatments were the same as those described previously^24^. For stress treatments, plants at the 5-leaf stage were transferred from hydroponic solution to fresh one supplemented with 15% PEG 6000 (osmotic stress), 500 µM CdSO_4_ (heavy metal stress) or pre-warmed at 42 °C (heat stress). The entire shoots and roots (treated and untreated) were independently collected at all different time points (0 h, 1 h30', 3 h, 6 h, 11 h and 24 h), immediately frozen in liquid nitrogen, and then stored at −80 °C till use. Three biological replicates were applied for all the treatments at every sampling time point.

### Candidate reference gene selection, PCR primer design

Among the sequenced genomes deposited in Phytozome, *Sorghum bicolor* and foxtail millet (*Setaria italica* L.) are the two species phylogenetically most closely related to *A. donax* ^23^. *A.donax* homologs of four common housekeeping genes from foxtail millet *TLF* (*Terminal Flower-like*), *Act 2* (*Actin 2*), *Tub α* (*Tubulin alpha*), and *EF-1 α* (*Elongation Factor 1 alpha*)^28^ were selected by BLASTN searches against the giant reed reference and water-stress transcriptomes^23, 24^. Analogously, two additional genes were identified in the *A. donax* transcriptomes based on stable expression across tissues (*RPN6* ^29^) or because already used as a reference gene in sorghum (*GAPDH*). Finally, the last two genes were selected directly from the giant reed transcriptome (*AC1* and *pDUF221* ^24^) based on their low coefficient of variation (CV) across organs/water stress conditions. The AC1 candidate had been previously chosen (Fu *et al*. 2016), because among the different *A. donax* transcripts coding for actin, it was the one with the lowest CV in the PEG-treated transcriptomes. The pDUF221 candidate was chosen exclusively based on the CV from the PEG-treated transcriptomes, where it ranked 5^th^ among the most stable transcripts for this stress (Supplementary Figure S5).

Primers were designed with Primer3Plus software (http://primer3plus.com/cgi-bin/dev/primer3plus.cgi) using the following parameters: length 18–25 bp (optimum 20), product size 75–200 bp, melting temperature 59–64 °C (optimum 60 °C); GC content 30–70% (optimum 50%). Primer pairs with free energy (dG) of dimer formation lower than -5 kcal/mol according to the PerlPrimer v1.1.21 software (http://perlprimer.sourceforge.net/) were discarded.

### Total RNA isolation and cDNA synthesis

Total RNA was isolated with the Spectrum Plant Total RNA Extraction Kit (Sigma) for shoots and the Rneasy® Plant Mini Kit (Qiagen) for roots, respectively. To assure complete absence of genomic DNA contaminations, extracted total RNA was treated with DNase I (Sigma-Aldrich) and checked on 1% agarose gel for integrity control. Concentration and quality of each sample were measured spectrophotometrically through the OD_260_/OD_280_ absorption ratio. First strand cDNA was reversed transcribed from 1 µg of total RNA primed with oligo-dT in a total reaction mixture of 20 µL using SuperScript® III Reverse Transcriptase (Life Technologies) according to the manufacture’s instruction.

### PCR amplification specificity and Quantitative Real Time PCR analyses

To assess the amplification specificity of each primer pair prior to qPCR analysis, PCR amplification was performed in a total volume of 10 µL containing 1 µl of 6-fold diluted cDNA (8 ng of starting RNA), 1x PCR Buffer, 100 nM dNTPs, 200 nM of each primer, 0.5 unit of *Taq* polymerase (Sigma) and 4.9 µl of H_2_O; The PCR programme was as follows: 8 min at 95 °C, 33 cycles of 40 s at 94 °C, 30 s at 60 °C and 20 s at 72 °C, with 5 min final extension at 72 °C. The PCR products were run on 2% agarose gel to check single amplification (Supplementary Figure S1A). The qPCR reaction was conducted by mixing 1 µL of 10-fold diluted cDNA (5 ng of starting RNA), 200 nM of each primer and 6.25 µL of Platinum® SYBR® Green qPCR SuperMix-UDG (Invitrogen) in a final volume of 12.5 µl. The programme for qRT-PCR in Bio-Rad C1000 Thermal Cycler was set as: 2 min at 50 °C, 2 min at 95 °C, 40 cycles of 15 s at 95 °C and 30 s at 60 °C (59 °C for target *DREB2A* gene). The melting curves were recorded after cycle 40 for every gene by constantly raising the temperature from 65 °C to 90 °C (Supplementary Figure S1B). A standard curve of qPCR reaction was generated from five points (four points for *pDUF221*gene and target gene *DREB2A*) of a 6-fold dilution series (10-fold dilution for *pDUF221* and *TLF*). The slope (S) of the standard curve was used to calculate the amplification efficiency (E) of each primer pair as follows: E = 10 ^(−1/S)^ (Supplementary Figure S2). Three technical replicates were used for each sample and every plate contained one No Template Control (NTC) well for each primer pair used. In order to compare different plates, in Bio-Rad CFX Manager software the baseline threshold was set at 329.82 and one control sample was used in every plate to check for Cq congruency. For the validation of the reference genes under cadmium stress, as *IspS* is under circadian regulation^50^, at each time point the relative expression was normalized with the untreated control at the same time.

### Data analyses with geNorm, NormFinder and BestKeeper

The analyses were conducted on six different datasets that comprise each a single combination of organ (shoots or roots) and indpendent stress treatment (osmotic, heavy metal or heat shock); in addition, one analysis was performed on the combined dataset comprising both organs in all stress conditions. This is necessary because the reference gene may vary depending on the experimental settings^7^. Three different Excel-based algorithms have been applied for data analysis. geNorm v3.5^10^ and NormFinder v0.953^11^ require relative input data, so the Cq values were converted with the formula 2^−ΔCt^ where ΔCt is the difference of each Cq value minus the lowest Cq value (highest expression level). BestKeeper^12^ instead, uses raw Cq values. geNorm calculates stability value (M) based on the average pairwise comparison with a stepwise exclusion of the highest M value (least stable gene). Further, geNorm calculates the number of genes needed for a reliable normalization considering the pairwise variation (V_n_/V_n+1_) between sequential normalization factors, NF_n_ and NF_n+1_. This number is optimal when the addition of one more reference gene does not significantly contribute to the variation of the normalization factor (NF_n+1_) or, as suggested, the value drops below 0.15. NormFinder uses an ANOVA-based algorithm to estimate intra- and inter- group variation for a given set of experiments, providing a rank where the most stable gene is the one with lowest stability (S) value. Moreover, NormFinder provides the best gene pair combination that minimizes the expression differences among subgroups, if subgroups are set. BestKeeper, differently from the other algorithms, does not provide a direct rank list but calculates standard deviation (SD [ ± Cq]) and coefficient of variation (CV [%Cq]) for each gene. We sorted the CV values to rank the genes from most stable (lowest CV value) to least stable (highest CV value).

## Data Availability

All data analysed during this study are included in this article (and its Supplementary Information files).

## Electronic supplementary material

Supplementary information
