# Supplementary material for: Selection of reference genes suitable for normalization of qPCR data under abiotic stresses in bioenergy crop Arundo donax L
Source: Sci Rep. 2017 Sep 6;7:10719. doi: 10.1038/s41598-017-11019-0 (PMC5587670; doi:10.1038/s41598-017-11019-0)
Supplement: Supplementary file 1 — Supplementary information [file 41598_2017_11019_MOESM1_ESM.pdf]

Selection of reference genes suitable for normalization of qPCR data under abiotic stresses in bioenergy crop *Arundo donax L.*

Michele Poli [1,2], Silvio Salvi [2], Mingai Li [1*], Claudio Varotto [1*].

[1] Department of Biodiversity and Molecular Ecology, Research and Innovation Centre, Fondazione Edmund Mach, Via E. Mach 1, 38010 S. Michele all'Adige (TN), Italy;

[2] Department of Agricultural Sciences, University of Bologna, Bologna, Italy.

[*] *Corresponding authors*: Mingai Li, mingai.li@fmach.it; Claudio Varotto, claudio.varotto@fmach.it

Supplementary tables

Gene	Species reference	Original accession No.	*Arundo donax* best hit	Score (bits)	E Value	Identities	*Arundo* primers F/R (5'-3')
**AC1**<br>Actin	*Sorghum bicolor*	Sobic.001G112600.1	Unigene036290	749	0.0	587/655 (89%)	TCTTGGCTTGCATTCTTGGG<br>TGGATTGCGAAGGCTGAGTAC
**Act2**<br>Actin2	*Setaria italica*	Seita.8G043100.1	Unigene057037	698	0.0	496/544 (91%), 347/399 (86%)	CGCATACGTGGCACTTGACT<br>GGGCATCTGAACCTCTCTGC
**EF-1α**<br>Elongation factor 1-alpha	*Setaria italica*	Seita.3G265600.1	Unigene076509	1049	0.0	643/681 (94%)	TGACTGTGCTGTGCTCATCA<br>GTTGCAGCAGCAGATCATCT
**GAPDH**<br>Glyceraldehyde-3-phosphate dehydrogenase 2	*Sorghum bicolor*	Sobic.007G025400.1	Unigene069707	1289	0.0	923/1014 (91%)	TGACAAGGAGAAGGCTGCTG<br>GAGCAAGGCAGTTTGTGGTG
**RPN6**<br>26S proteasome non-ATPase regulatory subunit 11	*Sorghum bicolor*	Sobic.006G092800.2	Unigene067565	1855	0.0	1212/1304 (92%)	CACACGACTAGCAGCTTTCAAG<br>TTCAAACGTCGGGAAGGTTG
**pDUF221**<br>Probable membrane protein DUF221-related Calcium-dependent channel	*Arundo donax*	N.A.	Unigene070087	N.A.	N.A.	N.A.	GACAAAGGAGTCAGCCGTCA<br>AACGTGCTTCGGACTTGGAT
**TLF**<br>Translation factor	*Setaria italica*	Seita.5G313000.1	Unigene076539	3640	0.0	2358/2532 (93%)	GACTTCATGGGTGGTGCTGA<br>TGTTTGTTGGGGGACTTGCT

TUB α<br>Alpha tubulin	*Setaria italica*	Seita.2G364600.1	Unigene068813	829	0.0	490/514 (95%), 440/498 (88%)	TACCAGCCACCCTCAGTTGT<br>AGTCGAACTTGTGGTCAATGC
**DREB2A***<br>Dehydration-Responsive Element Binding Protein 2	*Setaria italica*	Seita.3G281000.1	Unigene057213	416	e-115	327/366 (89%)	TCCAGCAGGTAGATCATCTCC<br>AGCAGGTTCGGTAATAGGCA
**IspS***<br>Isoprene synthase	*Populus canescens*	AJ294819.1	KX906604.1	N.A.	N.A.	N.A.	GAGGTTCCGTTGCATTTGAG<br>CAAGAGCAACATCTGTCCAC

Supplementary Table S1. Gene description and primers. The modified single bases or new primers are underlined. Asterisc (*) indicates the target genes for reference gene validation.

**Sample**	**pDUF221**	**TLF**	**AC1**	**Act2**	**TUB α**	**EF-1α**	**RPN6**	**GAPDH**
**Control_shoot_1**	26,84	24,18	23,00	22,49	22,81	22,02	25,04	19,53
**Control_shoot_2**	26,85	23,55	22,87	22,96	23,14	21,62	25,20	20,01
**Control_shoot_3**	27,99	24,20	23,27	23,71	23,76	22,14	25,65	20,08
**Control_root_1**	25,18	22,55	21,45	21,26	21,62	19,76	23,39	18,60
**Control_root_2**	25,20	21,44	21,85	20,93	21,37	19,02	23,20	18,23
**Control_root_3**	25,72	21,79	21,59	21,31	21,43	19,27	23,21	18,30
**Drought_shoot_1h30'_1**	26,19	22,68	22,40	22,27	21,96	20,51	24,00	19,09
**Drought_shoot_1h30'_2**	26,38	23,46	22,86	22,76	23,19	21,19	24,42	19,20
**Drought_shoot_1h30'_3**	26,41	23,76	23,58	23,33	23,53	21,53	24,72	19,79
**Drought_shoot_3h_1**	25,77	24,01	23,55	23,07	23,48	21,56	24,31	19,55
**Drought_shoot_3h_2**	25,58	24,06	23,44	23,59	23,60	21,86	24,79	20,11
**Drought_shoot_3h_3**	25,83	23,15	22,58	22,90	23,10	20,71	24,27	19,12
**Drought_shoot_6h_1**	25,25	23,96	23,42	23,56	22,93	21,51	24,97	19,85
**Drought_shoot_6h_2**	24,73	23,29	24,03	23,30	22,93	21,65	24,77	19,55
**Drought_shoot_6h_3**	25,14	24,15	24,56	23,95	23,57	22,09	24,83	20,06
**Drought_shoot_11h_1**	25,02	24,05	24,39	23,75	23,92	21,38	24,99	19,47
**Drought_shoot_11h_2**	24,58	24,22	24,01	23,22	23,35	21,31	24,82	19,35
**Drought_shoot_11h_3**	24,94	24,45	24,99	23,66	23,90	21,92	25,01	19,71

**Drought_shoot_24h_1**	26,14	24,93	24,90	22,93	24,59	22,90	25,28	19,30
**Drought_shoot_24h_2**	26,11	24,65	23,68	22,36	23,56	22,23	25,13	19,31
**Drought_shoot_24h_3**	25,62	24,26	23,36	21,95	23,21	21,76	24,83	19,08
**Drought_root_1h30'_1**	25,68	22,65	22,15	21,96	22,62	19,65	24,03	19,11
**Drought_root_1h30'_2**	25,56	22,18	21,90	21,52	22,04	19,35	23,80	18,69
**Drought_root_1h30'_3**	25,58	21,96	21,66	21,74	21,81	19,62	23,53	18,57
**Drought_root_3h_1**	24,98	22,64	24,15	22,24	23,45	19,48	23,87	18,84
**Drought_root_3h_2**	25,59	22,78	23,38	22,24	23,21	19,42	23,93	18,66
**Drought_root_3h_3**	26,05	23,41	23,74	23,01	23,75	20,75	24,55	19,24
**Drought_root_6h_1**	24,80	23,06	23,41	22,93	23,14	20,24	23,72	19,07
**Drought_root_6h_2**	25,16	22,94	24,94	22,96	23,89	20,50	24,14	18,59
**Drought_root_6h_3**	24,68	23,40	25,14	22,82	24,09	20,77	24,32	19,38
**Drought_root_11h_1**	23,83	21,47	24,27	22,36	23,36	20,04	24,03	18,49
**Drought_root_11h_2**	24,17	21,58	24,18	22,25	23,14	19,86	23,68	18,76
**Drought_root_11h_3**	24,39	22,31	23,91	22,34	23,40	20,28	24,18	19,42
**Drought_root_24h_1**	24,59	21,38	22,39	21,55	22,12	19,42	23,53	18,15
**Drought_root_24h_2**	25,10	21,77	22,13	21,86	22,21	19,19	23,26	18,25
**Drought_root_24h_3**	24,83	21,82	22,31	23,38	22,12	19,51	23,48	18,27
**Cadmium_shoot_1h30'_1**	26,26	22,10	22,11	21,71	21,72	21,02	24,87	19,14
**Cadmium_shoot_1h30'_2**	26,32	21,71	21,85	21,43	21,97	20,21	24,78	19,21
**Cadmium_shoot_1h30'_3**	26,75	21,94	21,76	21,75	22,12	20,34	24,79	19,55
**Cadmium_shoot_3h_1**	26,96	23,16	23,20	22,82	23,25	21,44	25,41	20,40
**Cadmium_shoot_3h_2**	26,54	22,86	23,28	22,61	22,77	21,73	25,29	19,80
**Cadmium_shoot_3h_3**	26,61	22,57	22,97	22,42	22,35	21,58	25,08	19,48
**Cadmium_shoot_6h_1**	25,44	21,95	22,94	22,72	22,37	20,65	24,79	19,78
**Cadmium_shoot_6h_2**	25,52	21,24	21,66	21,96	21,56	19,56	24,16	18,84
**Cadmium_shoot_6h_3**	25,53	22,31	23,20	22,98	22,48	21,58	25,20	19,87
**Cadmium_shoot_11h_1**	25,55	22,08	21,59	21,98	21,56	20,69	24,33	18,11
**Cadmium_shoot_11h_2**	25,51	22,43	22,24	22,24	21,90	21,29	24,71	19,36
**Cadmium_shoot_11h_3**	25,51	22,01	22,17	21,91	21,75	21,10	24,90	19,13
**Cadmium_shoot_24h_1**	26,21	22,16	21,82	21,12	21,92	21,19	24,78	19,01

**Cadmium_shoot_24h_2**	26,23	22,30	21,70	21,04	22,21	21,15	24,59	19,05
**Cadmium_shoot_24h_3**	26,85	23,66	22,21	22,08	22,85	22,39	25,27	19,65
**Cadmium_root_1h30'_1**	26,05	21,92	22,44	22,25	22,37	19,32	23,66	17,29
**Cadmium_root_1h30'_2**	25,58	22,13	22,94	22,53	22,32	19,62	23,61	17,62
**Cadmium_root_1h30'_3**	25,50	21,96	23,57	22,25	23,19	20,20	24,11	17,83
**Cadmium_root_3h_1**	25,51	22,60	24,15	22,68	23,19	20,92	24,42	18,31
**Cadmium_root_3h_2**	25,40	22,26	22,84	21,74	22,12	19,52	23,52	17,79
**Cadmium_root_3h_3**	25,63	22,76	24,33	22,17	23,48	19,88	23,99	18,31
**Cadmium_root_6h_1**	25,36	22,57	23,58	22,40	22,81	20,09	24,36	17,94
**Cadmium_root_6h_2**	25,50	22,06	22,80	21,75	21,96	19,28	23,71	17,14
**Cadmium_root_6h_3**	25,60	22,15	23,53	22,74	22,80	19,50	24,12	18,03
**Cadmium_root_11h_1**	25,87	22,17	23,68	22,36	23,15	19,92	23,45	17,06
**Cadmium_root_11h_2**	25,77	23,26	25,07	23,08	24,28	20,45	24,36	18,12
**Cadmium_root_11h_3**	25,62	22,05	23,44	21,92	22,94	19,05	23,12	16,16
**Cadmium_root_24h_1**	26,09	21,83	23,20	22,18	22,90	19,29	23,56	17,24
**Cadmium_root_24h_2**	26,17	22,68	24,40	22,03	23,22	19,96	23,47	17,49
**Cadmium_root_24h_3**	25,97	22,21	23,94	21,87	23,11	19,59	23,19	17,25
**HeatShock_shoot_1h30'_1**	25,79	23,96	24,55	21,93	24,18	21,99	25,12	18,53
**HeatShock_shoot_1h30'_2**	25,42	23,40	23,58	21,12	23,32	21,27	24,26	17,82
**HeatShock_shoot_1h30'_3**	25,65	23,66	24,24	22,08	24,04	21,83	24,92	18,26
**HeatShock_shoot_3h_1**	24,17	22,88	24,69	22,20	23,11	21,34	24,69	18,64
**HeatShock_shoot_3h_2**	24,29	23,22	24,24	22,13	23,05	21,20	24,76	18,78
**HeatShock_shoot_3h_3**	24,54	23,23	24,58	22,36	23,23	21,43	24,95	18,88
**HeatShock_shoot_6h_1**	24,09	21,85	23,67	22,61	22,15	20,88	24,33	19,09
**HeatShock_shoot_6h_2**	24,28	22,21	23,93	22,91	22,20	20,91	24,68	19,28
**HeatShock_shoot_6h_3**	24,68	22,89	23,86	22,99	22,38	21,17	24,68	19,65
**HeatShock_shoot_11h_1**	24,77	22,51	23,21	22,35	22,49	20,73	24,66	19,20
**HeatShock_shoot_11h_2**	24,79	22,27	22,99	22,09	22,11	20,65	24,49	19,19
**HeatShock_shoot_11h_3**	24,78	22,61	23,15	22,46	22,52	21,05	24,66	19,34
**HeatShock_shoot_24h_1**	25,78	23,27	23,14	21,41	23,01	21,45	24,82	19,05
**HeatShock_shoot_24h_2**	26,08	23,79	23,43	21,63	23,21	21,82	24,80	19,31

**HeatShock_shoot_24h_3**	26,25	23,57	23,24	21,56	22,76	21,51	24,64	19,06
**HeatShock_root_1h30'_1**	24,86	22,48	25,88	23,12	23,30	19,91	24,72	17,83
**HeatShock_root_1h30'_2**	24,62	22,48	26,22	21,85	24,37	20,17	24,57	17,84
**HeatShock_root_1h30'_3**	24,52	22,74	26,07	21,47	23,58	20,40	24,54	17,90
**HeatShock_root_3h_1**	24,07	23,71	26,05	22,12	23,47	20,90	24,67	18,80
**HeatShock_root_3h_2**	23,57	23,56	26,81	21,96	23,84	20,40	24,67	18,90
**HeatShock_root_3h_3**	23,55	23,74	27,08	21,66	24,28	20,49	24,53	18,80
**HeatShock_root_6h_1**	23,22	24,20	28,58	23,02	24,55	21,40	25,25	20,10
**HeatShock_root_6h_2**	23,32	23,40	26,73	22,18	24,31	20,68	24,79	19,50
**HeatShock_root_6h_3**	23,80	23,07	26,82	22,30	24,50	20,50	24,78	18,95
**HeatShock_root_11h_1**	24,16	24,06	26,58	22,46	24,17	20,78	25,30	19,48
**HeatShock_root_11h_2**	23,26	23,31	26,43	22,13	23,53	20,09	24,87	19,14
**HeatShock_root_11h_3**	24,19	23,41	24,84	21,69	23,06	20,31	25,08	18,93
**HeatShock_root_24h_1**	23,32	23,20	25,63	21,33	23,09	20,33	24,23	19,10
**HeatShock_root_24h_2**	23,48	23,55	25,53	21,19	23,30	20,46	24,53	19,30
**HeatShock_root_24h_3**	23,32	23,08	25,23	21,33	23,06	20,29	24,20	19,15

Supplementary Table S2. Raw Cq values for each candidate reference gene for specific samples (i.e. combination of stress, tissue and stress length).

	**pDUF221**	**TLF**	**AC1**	**Act2**	**TUB α**	**EF-1α**	**RPN6**	**GAPDH**
**Average**	25,276	22,822	23,703	22,278	22,939	20,616	24,398	18,840
**SD**	0,962625	0,836003	1,478618	0,697158	0,815829	0,898593	0,61424	0,796856
**CV**	3,81%	3,66%	6,24%	3,13%	3,56%	4,36%	2,52%	4,23%
**Median**	25,50	22,71	23,49	22,24	23,08	20,65	24,55	19,05
**25% Percentile**	24,63	22,14	22,64	21,75	22,21	19,89	23,99	18,27
**75% Percentile**	25,95	23,45	24,51	22,82	23,48	21,37	24,81	19,38
**Percentile Difference**	1,31	1,32	1,87	1,07	1,27	1,48	0,82	1,10

Supplementary Table S3. Cq details. SD = standard deviation, CV (coefficient of variation).

Rank	All						Osmotic					
	GeNorm		NormFinder		BestKeeper		GeNorm		NormFinder		BestKeeper	
	Gene	Value	Gene	Value	Gene	Value	Gene	Value	Gene	Value	Gene	Value
1	EF-1α RPN6	0.493	RPN6	0.134	RPN6	2,03±0,49	TLF EF-1α	0.407	RPN6	0.062	GAPDH	2,48±0,47
2	GAPDH	0.493	EF-1α	0.145	Act2	2,43±0,54	RPN6	0.474	GAPDH	0.209	RPN6	2,33±0,57
3	TLF	0.581	TLF	0.210	GAPDH	3,34±0,63	GADPH	0.513	TUB α	0.291	TUB α	2,77±0,64
4	Act2	0.646	TUB α	0.229	TUB α	2,80±0,64	Act2	0.563	Act2	0.311	pDUF221	2,54±0,65
5	TUB α	0.711	Act2	0.231	TLF	3,17±0,73	TUB α	0.593	TLF	0.345	Act2	2,94±0,66
6	pDUF221	0.752	GAPDH	0.246	EF-1α	3,65±0,75	AC1	0.666	EF-1α	0.348	AC1	3,65±0,85
7	AC1	0.885	AC1	0.595	pDUF221	3,08±0,78	pDUF221	0.758	AC1	0.579	TLF	3,83±0,89
8			pDUF221	0.646	AC1	4,74±1,13			pDUF221	0.648	EF-1α	4,61±0,95

Rank	Heavy metal						Heat shock					
	GeNorm		NormFinder		BestKeeper		GeNorm		NormFinder		BestKeeper	
	Gene	Value	Gene	Value	Gene	Value	Gene	Value	Gene	Value	Gene	Value
1	EF-1α RPN6	0.404	TLF	0.234	Act2	2,19±0,49	TLF RPN6	0.488	RPN6	0.167	RPN6	1,39±0,34
2	pDUF221	0.586	Act2	0.275	pDUF221	1,94±0,50	EF-1α	0.527	TLF	0.196	GAPDH	2,44±0,46
3	TLF	0.615	pDUF221	0.306	TLF	2,30±0,52	GADPH	0.599	Act2	0.332	Act2	2,36±0,52
4	Act2	0.657	RPN6	0.307	TUB α	2,67±0,60	Act2	0.624	EF-1α	0.335	TLF	2,46±0,57
5	TUB α	0.694	TUB α	0.398	RPN6	2,64±0,64	TUB α	0.686	TUB α	0.340	EF-1α	2,89±0,60
6	GADPH	0.755	EF-1α	0.417	AC1	3,29±0,75	pDUF221	0.850	GAPDH	0.385	TUB α	2,83±0,66
7	AC1	0.832	GAPDH	0.624	EF-1α	4,14±0,85	AC1	1.081	pDUF221	0.936	pDUF221	3,64±0,90
8			AC1	0.677	GAPDH	4,83±0,90			AC1	1.186	AC1	5,84±1,44

Supplementary table S4. Expression stability ranks calculated with GeNorm, NormFinder and BestKeeper in shoot and root together for each single stress and their combination (referred as “All”).

Supplementary figures

A

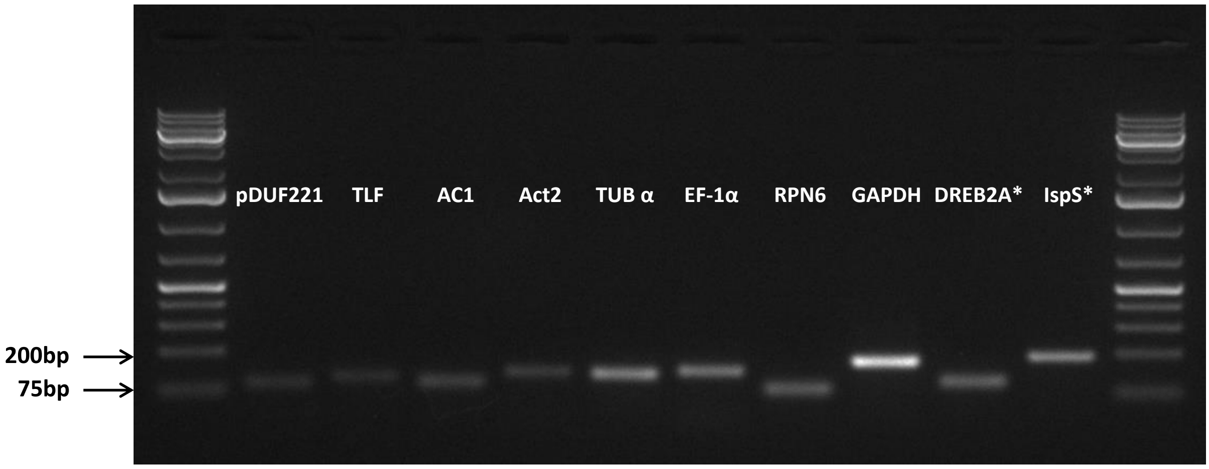

B

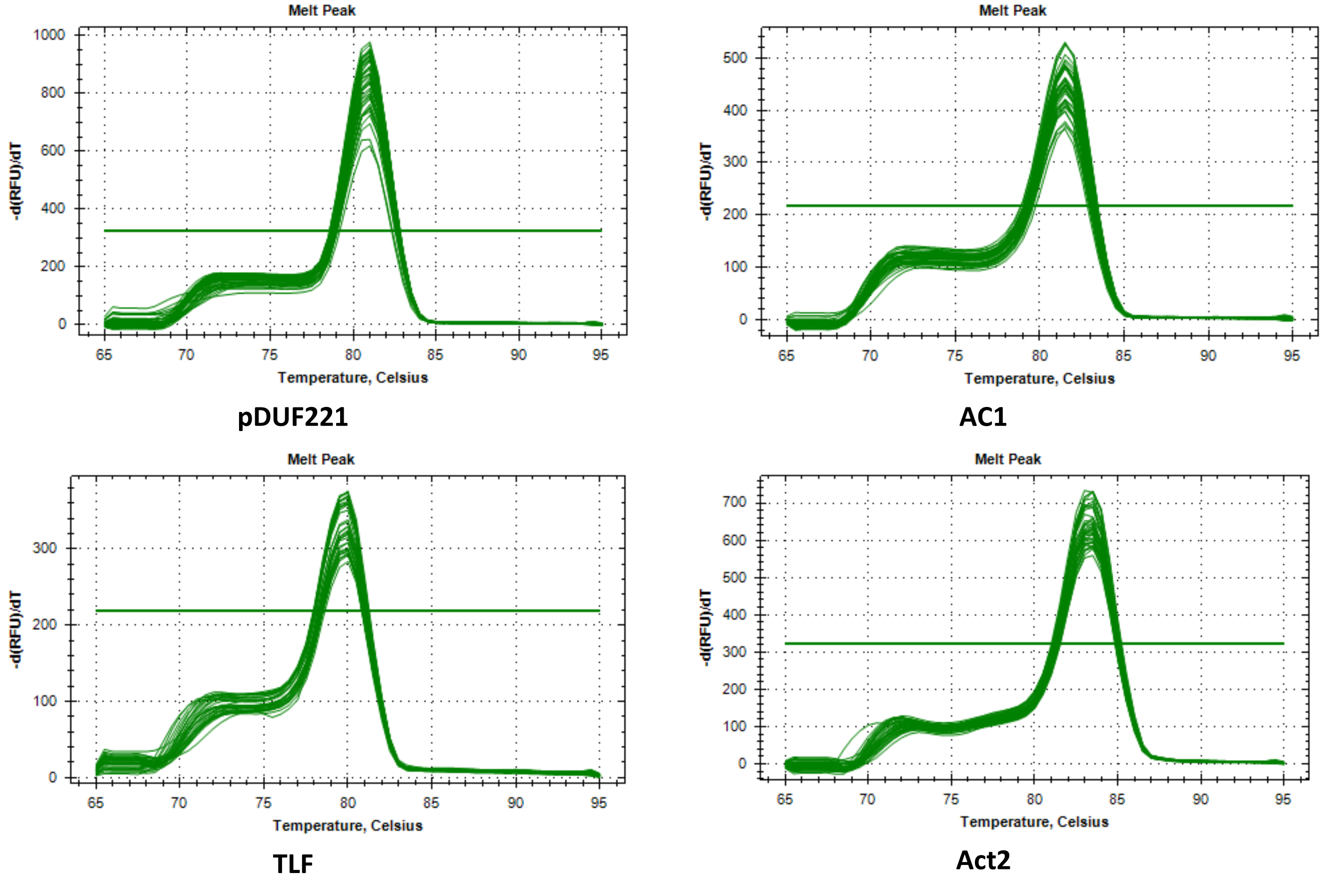

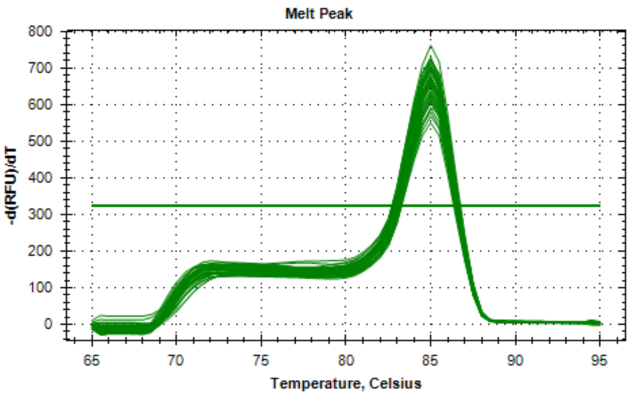

TUB α

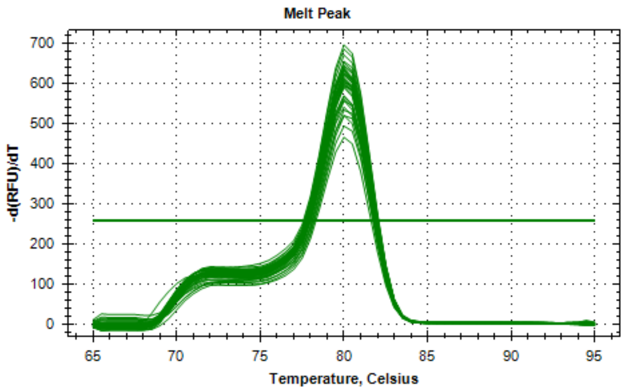

RPN6

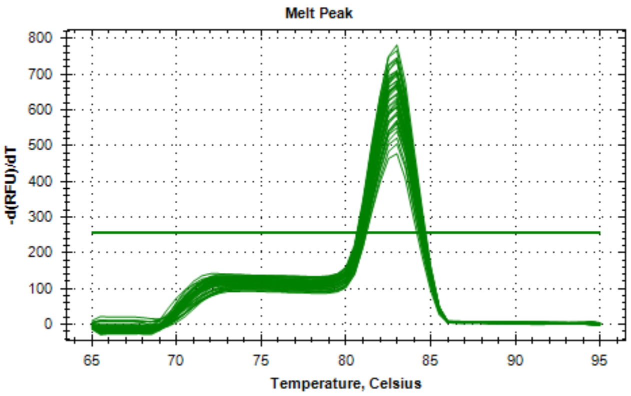

EF-1α

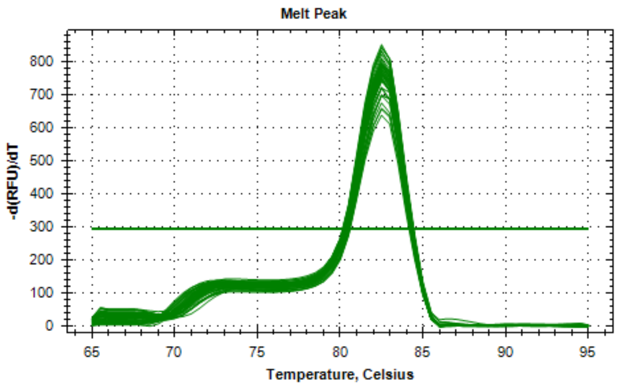

GAPDH

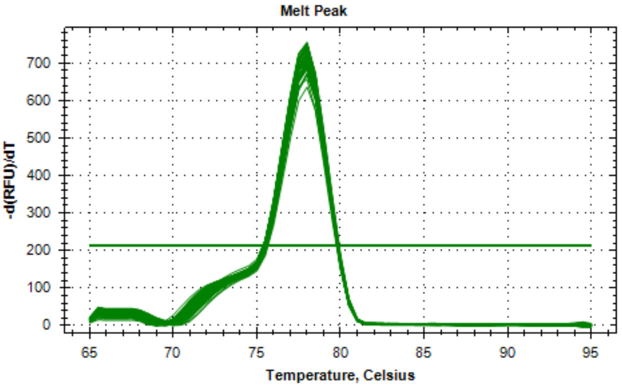

DREB2A*

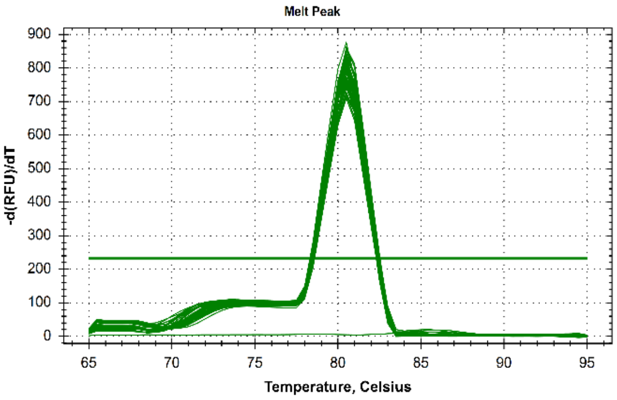

IspS*

Supplementary Figure S1. Amplicon sizes visualized on 2% agarose gel (A) and melting curves of eight candidate reference genes (B). Target genes used for reference gene validation are indicated by “*”.

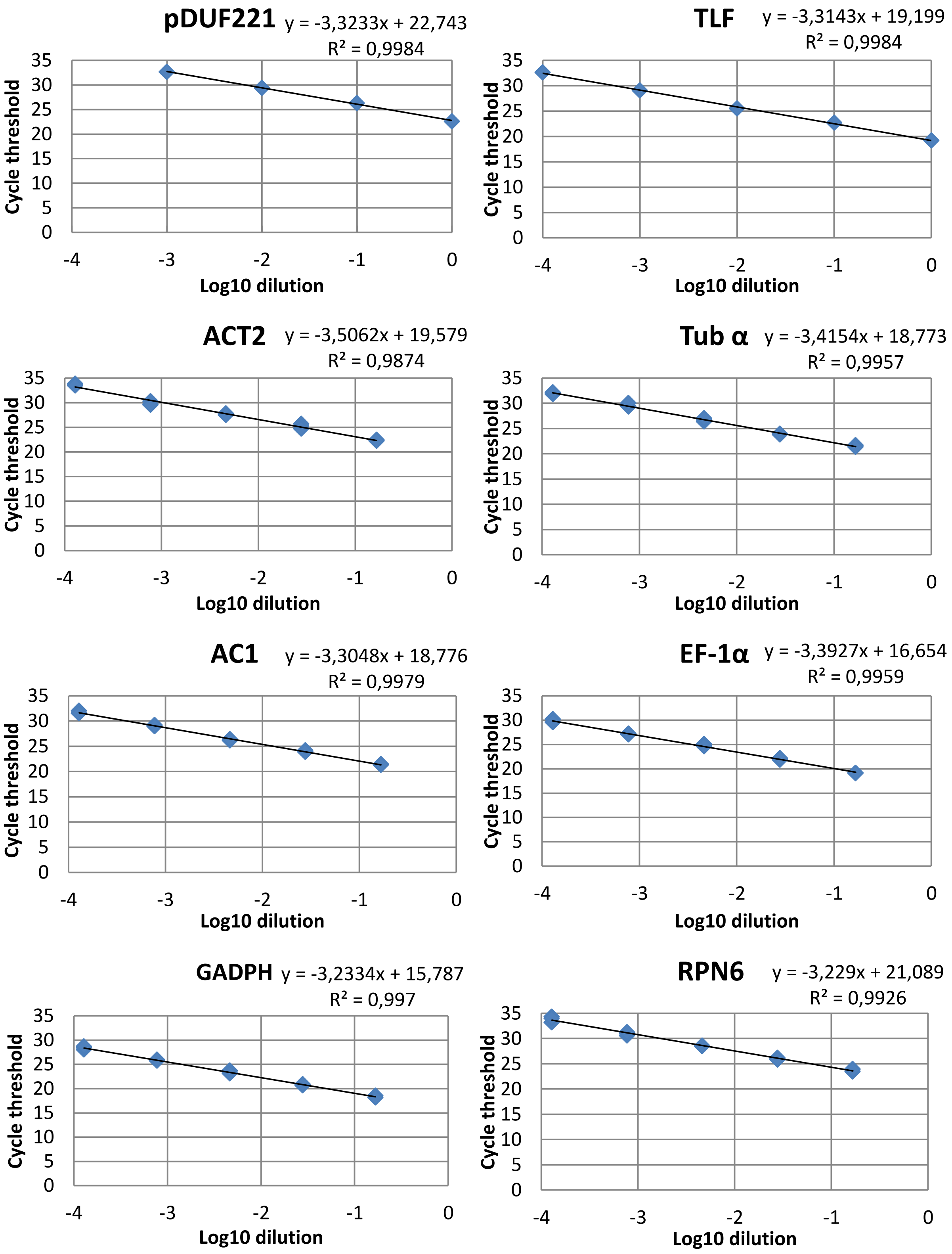
pDUF221
y = -3,3233x + 22,743
R² = 0,9984
TLF
y = -3,3143x + 19,199
R² = 0,9984
ACT2
y = -3,5062x + 19,579
R² = 0,9874
Tub α
y = -3,4154x + 18,773
R² = 0,9957
AC1
y = -3,3048x + 18,776
R² = 0,9979
EF-1α
y = -3,3927x + 16,654
R² = 0,9959
GADPH
y = -3,2334x + 15,787
R² = 0,997
RPN6
y = -3,229x + 21,089
R² = 0,9926
Cycle threshold
Log10 dilution
35
30
25
20
15
10
5
0
-4
-3
-2
-1
0

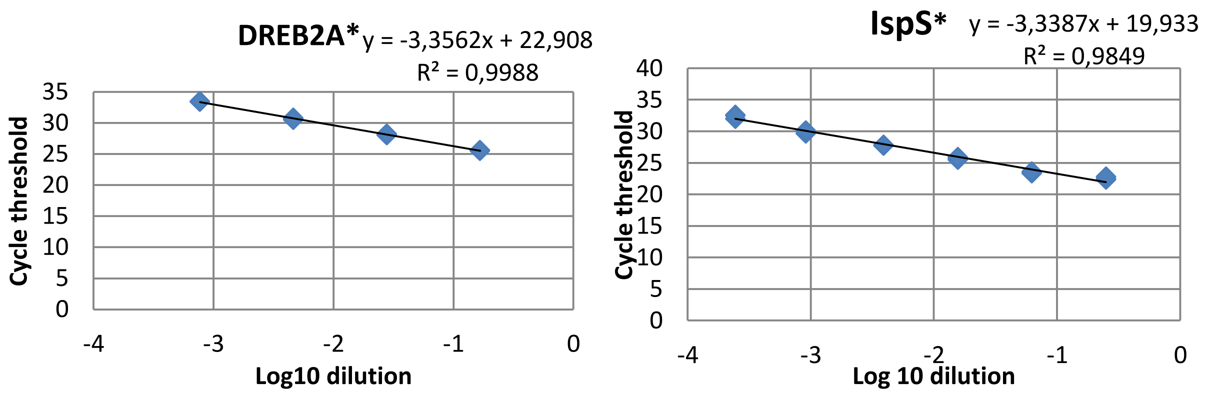

Supplementary Figure S2. Standard curves for eight candidate reference genes. Curves are made with a serial six-fold dilution. Blue squares indicate the three technical replicates for each dilution step. The genes used for reference gene validation are indicated by “*”.

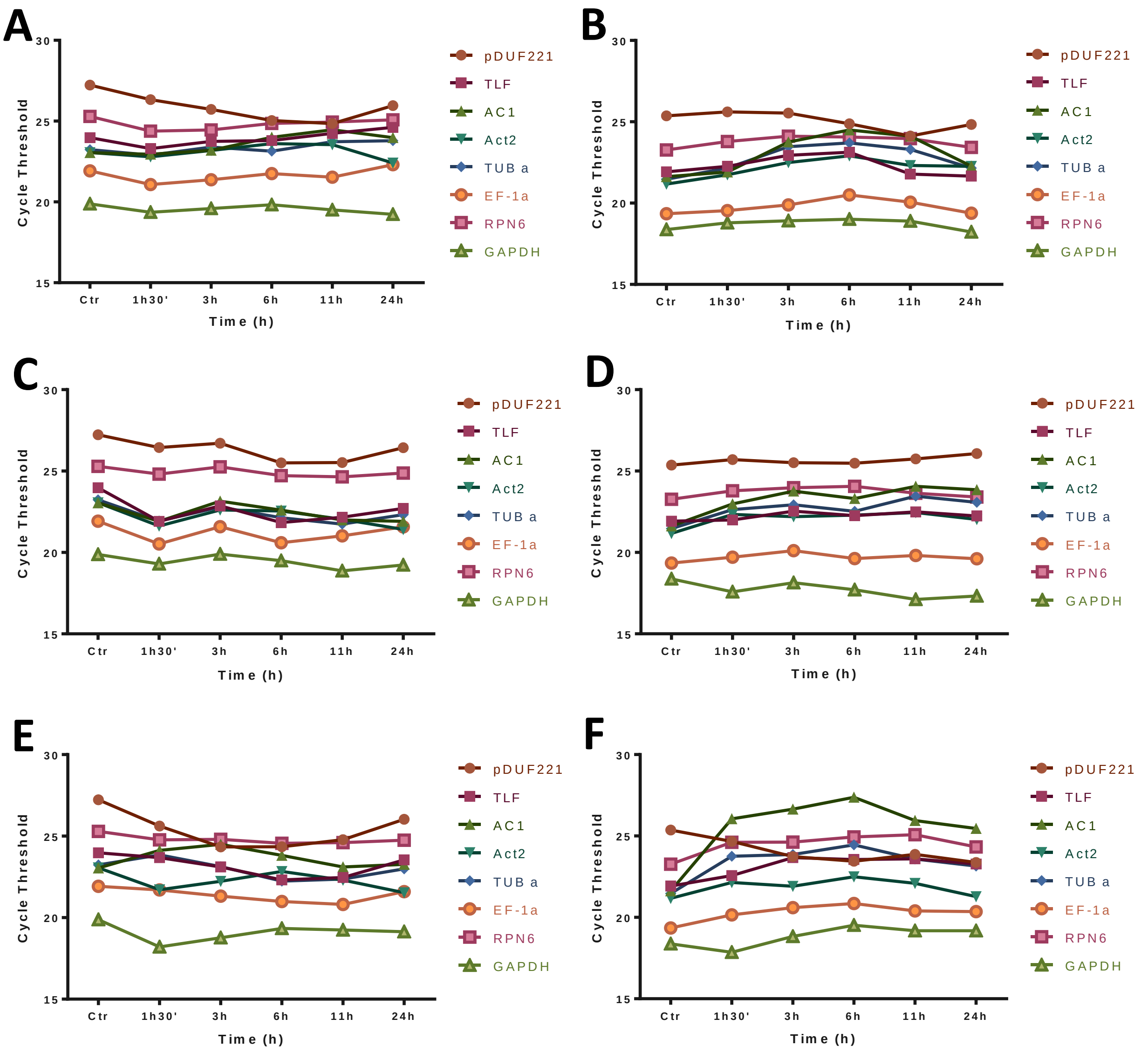

Supplementary Figure S3. Expression profile of eight candidate genes divided by stress and tissues:

Osmotic stress in shoot (A) and root (B); Heavy metal stress in shoot (C) and root (D); Heat shock stress in shoot (E) and root (F).

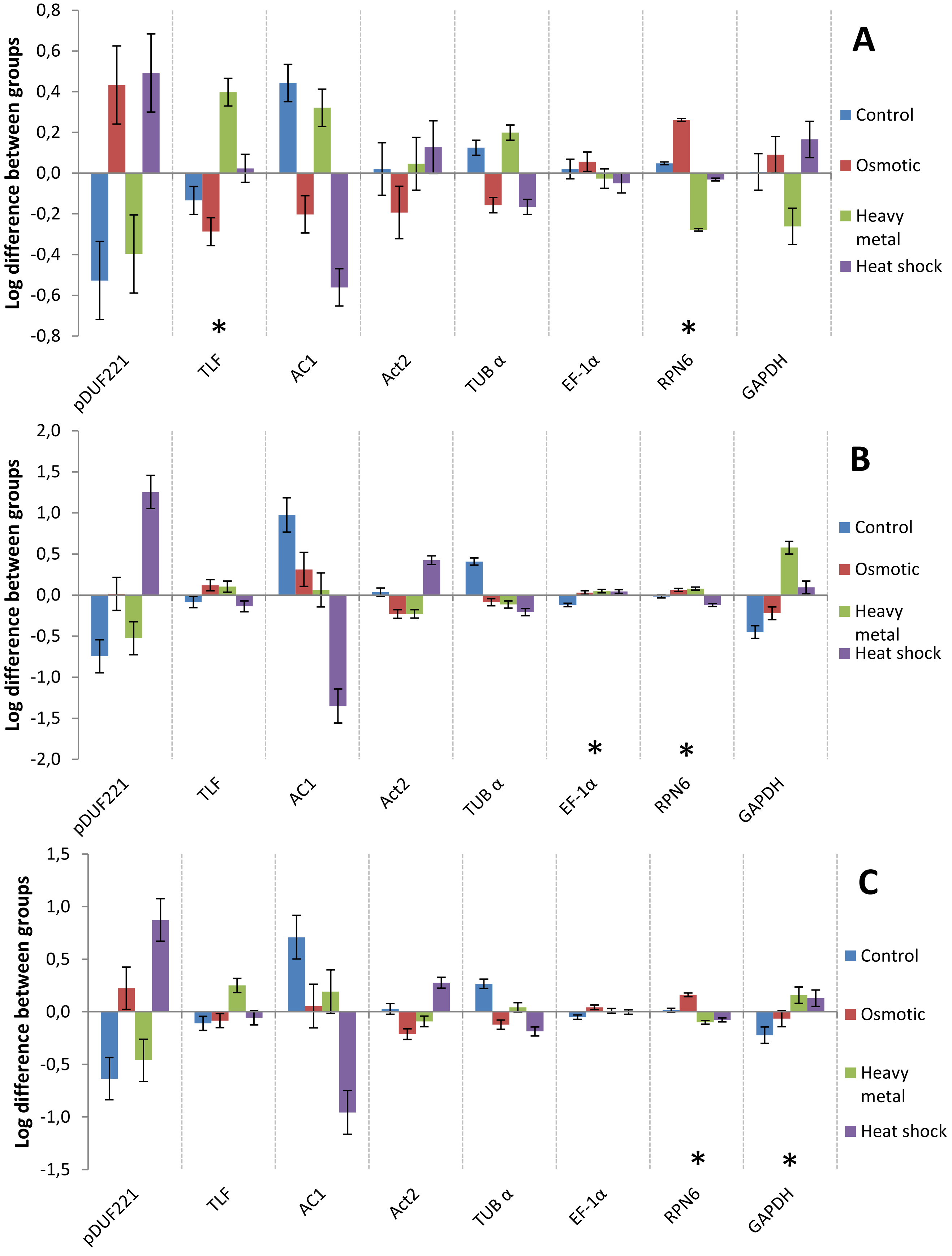

Supplementary Figure S4. NormFinder intergroup variation. Dataset was divided in shoot (A), root (B),

shoot + root (C) and input in NormFinder algorithm with four subgroups: control plus three stresses (osmotic, heavy metal and heat shock). Bars represent the intergroup variation respect to the average and vertical lines the mean of intragroup variation, asterisc (*) indicates the best combination of two genes.

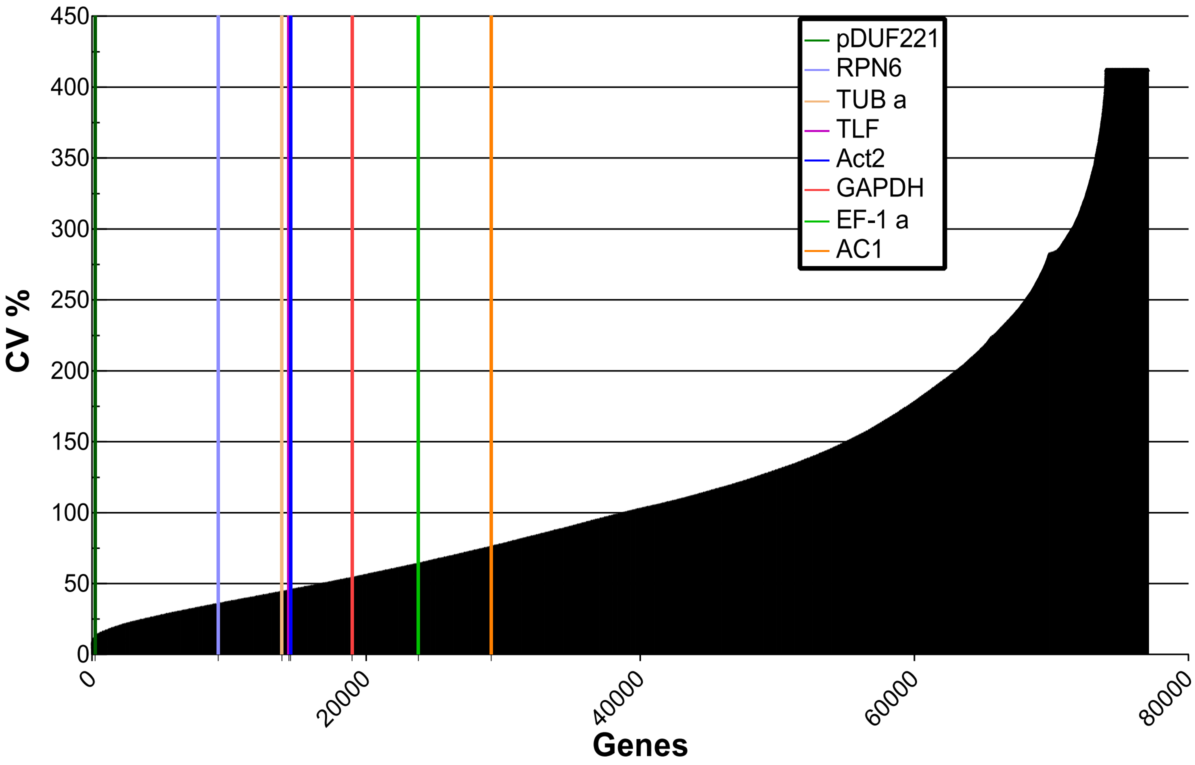

Supplementary Figure S5. Distribution of expression coefficients of variation (CV) in PEG-treated *A. donax* transcriptomes. The CV of all the transcripts analyzed in Fu et al. (2016) are reported. The colored bars indicate the eight transcripts used in this study as candidate reference genes. Note to the very left (close to the y-axis) the pDUF221 transcript, ranking 5th among all analyzed transcripts in terms of CV (the smaller the CV value, the more stable is the expression of the transcript in the condition tested).
